# Palmitoylethanolamide attenuates neurodevelopmental delay and early hippocampal damage following perinatal asphyxia in rats

**DOI:** 10.3389/fnbeh.2022.953157

**Published:** 2022-08-25

**Authors:** Maria I. Herrera, Lucas D. Udovin, Tamara Kobiec, Nicolas Toro-Urrego, Carlos F. Kusnier, Rodolfo A. Kölliker-Frers, Juan P. Luaces, Matilde Otero-Losada, Francisco Capani

**Affiliations:** ^1^Centro de Investigaciones en Psicología y Psicopedagogía, Facultad de Psicología, Pontificia Universidad Católica Argentina, Buenos Aires, Argentina; ^2^Centro de Altos Estudios en Ciencias Humanas y de la Salud, Universidad Abierta Interamericana, Consejo Nacional de Investigaciones Científicas y Técnicas, Buenos Aires, Argentina; ^3^Instituto de Ciencias Biomédicas, Facultad de Ciencias de la Salud, Universidad Autónoma de Chile, Santiago, Chile

**Keywords:** PEA, palmitoylethanolamide, perinatal asphyxia, neuroprotection, hippocampal CA1 area, reflexes, neurodevelopmental disorder (NDD)

## Abstract

Impaired gas exchange close to labor causes perinatal asphyxia (PA), a neurodevelopmental impairment factor. Palmitoylethanolamide (PEA) proved neuroprotective in experimental brain injury and neurodegeneration models. This study aimed to evaluate PEA effects on the immature-brain, i.e., early neuroprotection by PEA in an experimental PA paradigm. Newborn rats were placed in a 37°C water bath for 19 min to induce PA. PEA 10 mg/kg, s.c., was administered within the first hour of life. Neurobehavioral responses were assessed from postnatal day 1 (P1) to postnatal day 21 (P21), recording the day of appearance of several reflexes and neurological signs. Hippocampal CA1 area ultrastructure was examined using electron microscopy. Microtubule-associated protein 2 (MAP-2), phosphorylated high and medium molecular weight neurofilaments (pNF H/M), and glial fibrillary acidic protein (GFAP) were assessed using immunohistochemistry and Western blot at P21. Over the first 3 weeks of life, PA rats showed late gait, negative geotaxis and eye-opening onset, and delayed appearance of air-righting, auditory startle, sensory eyelid, forelimb placing, and grasp reflexes. On P21, the hippocampal CA1 area showed signs of neuronal degeneration and MAP-2 deficit. PEA treatment reduced PA-induced hippocampal damage and normalized the time of appearance of gait, air-righting, placing, and grasp reflexes. The outcome of this study might prove useful in designing intervention strategies to reduce early neurodevelopmental delay following PA.

## Introduction

Transient interruption of oxygen supply close to delivery causes an obstetrical complication known as perinatal asphyxia (PA) (Adcock and Papile, 2008). The estimated incidence of this life-threatening complication ranges from 1 to 8 up to 26 per 1,000 live births in developed and developing countries, respectively (Douglas-Escobar and Weiss, 2015). Neonatal care advances are so far unsuccessful in overcoming the impact of PA, which increases neonatal mortality, neurological morbidity, and neurodevelopmental disorders (NDDs) (Weitzdoerfer et al., 2004; Herrera-Marschitz et al., 2014). The extensively used Bjelke’s experimental model (Bjelke et al., 1991) has allowed to study PA neuropathological and behavioral effects (Barkhuizen et al., 2017). We have reported long-term PA-induced deficits (Capani et al., 2009; Galeano et al., 2011; Saraceno et al., 2010, 2012; Muñiz et al., 2014). We observed behavioral alterations 1 month after experimental PA (Saraceno et al., 2016; Herrera et al., 2018), though little is known about the early impact of the perinatal insult (Horvath et al., 2015; Barkhuizen et al., 2017). Hence, we examined body weight gain and several signs of neurological maturation in asphyctic rats throughout the first 3 weeks of life, corresponding to the first 3 years of human development (Clancy et al., 2007; Semple et al., 2013), a critical period for neurotypical and aberrant neurodevelopment (Meredith, 2015). Developmental reflex testing concerns human infants, while their evaluation in rats provides a translational expression of perinatal injuries, offering genuine developmental traits (Moser, 2001; Nguyen et al., 2017).

In clinical settings, therapeutic hypothermia (TH) offers partial neuroprotection (Blanco et al., 2011). Contrary to expectations, neuroprotective agents have not shown synergism combined with TH (Cilio and Ferriero, 2010, Azzopardi et al., 2016; Rüegger et al., 2018) and are expensive for developing countries with increasing PA incidence. Studying the effect of endogenous compounds becomes imperative (Tagin et al., 2015). Palmitoylethanolamide (PEA), the ethanolamide of palmitic (hexadecanoic) acid (Guida et al., 2017), is a pro-homeostatic compound (Petrosino et al., 2010; Petrosino and Di Marzo, 2017), abundant in the human and rodent brains (Di Marzo, 1998; Maccarrone and Finazzi-Agró, 2002, Maccarrone and Finazzi-Agró, 2003). We have reported that PEA treatment (10 mg/kg, within the first hour of life) attenuated cytoskeletal alterations in CA1 hippocampal neurons and improved behavioral outcomes 1 month after PA (Herrera et al., 2018). The present study intended to expand these results, describing PEA effects on neurodevelopment during the first weeks of life and the corresponding changes in the CA1 hippocampal area at P21. The CA1 area is particularly vulnerable to experimental PA (Petito and Pulsinelli, 1984; Pulsinelli, 1985; van de Berg et al., 2000; Li et al., 2019), and the hippocampus is damaged in children with NDDs and learning disabilities (Li et al., 2019). Knowledge of the early effects of PEA treatment is expected to help design early intervention strategies for the developing injured brain.

## Materials and methods

The experimental protocol was approved by the Institutional Animal Care and Use Committee of the University of Buenos Aires (CICUAL#4091/04). The experiments were conducted following the principles of the Guide for the Care and Use of Laboratory Animals (Animal Welfare Assurance, A-3033-01/protocol#S01084).

Only original figures are included in this manuscript. Some of them are cropped for space limitations and shown in full as Supplementary material.

### Animals

Twenty pregnant *Sprague-Dawley* rats from the central vivarium of the School of Veterinary Sciences of the University of Buenos Aires arrived at our local vivarium for environmental adaptation 1 week before delivery.

### Housing conditions

Animals were housed in individual cages at constant 21 ± 2°C temperature and 65 ± 5% humidity conditions with free access to food and tap water. Lights went on at 7 a.m., with 12:12 h light: dark cycles (Galeano et al., 2011).

### Induction of perinatal asphyxia

Rat pups were subjected to PA using Bjelke et al.’s original model modified in our laboratory (Bjelke et al., 1991; Capani et al., 2009). This experimental paradigm induces severe asphyxia by submerging rat pups immediately upon delivery in a water bath at 37°C. After 19 min, intermittent tactile stimulation is given until regular breathing restoration (Saraceno et al., 2010).

### Neuroprotection protocol

Within the first hour of life, 75 male rat pups were injected with either vehicle (VHI, 1:1:8 solution of DMSO, Tween 80 and NaCl) or PEA 10 mg/kg (Herrera et al., 2018). Only male pups were used to avoid confounding variables due to estrogens neuroprotective properties (Saraceno et al., 2010). Four experimental groups were studied: rats subjected to PA injected with VHI (PA-VHI, *n* = 19), rats born vaginally (control, CTL) injected with VHI (CTL-VHI, *n* = 21), rats subjected to PA injected with PEA (PA-PEA group, *n* = 17), and rats born vaginally injected with PEA (CTL-PEA group, *n* = 18). As this model includes euthanasia administration to the mothers, rat pups in all the experimental groups were placed by surrogate mothers, which had delivered vaginally in the previous 24 h. Rats were identified according to the group and placed in the respective litters (Udovin et al., 2020).

### Neurobehavioral development examination

Neurodevelopment was assessed from P1 to P21, e.g., during the first 3 weeks of life, between 12:00 and 15:00 p.m. Pups (*N* = 75) underwent daily weight control and testing of reflexes and signs, symptomatic of nervous system maturation (Kiss et al., 2009). The experimenter was blind to the groups, i.e., unaware of rat treatment.

• Surface righting reflex: pups were placed in the supine position. Time (seconds) to turn over to the prone position placing all four paws on the surface was recorded daily.
• Air-righting reflex: pups were dropped head down onto a bed of shavings from a height of 50 cm. The first day of landing on four paws was recorded (postnatal day of appearance).
• Gait: pups were placed at the center of a 13 cm diameter white paper circle. The test ended if the rat did not leave the circle after the first 30 s. Postnatal day of gait appearance, e.g., the first day the rat moved off the circle with both forelimbs was recorded. Thereafter, test performance was recorded, in seconds, daily.
• Forelimb placing reflex: the back of the forepaw of a suspended pup was touched with the bench edge. The first day of placing the paws on the table was recorded.
• Forelimb grasp reflex: forelimbs were touched with a thin rod. The first day of grasping onto the rod was recorded.
• Negative geotaxis: pups were placed head down, hindlimbs in the middle of a 45° inclined 30 cm long grid. The test was ended if the rat did not turn round, climbed up the board with their forelimbs, and reached the upper rim within the first 30 s. The first day the rat so did was recorded as the postnatal day of appearance. Thereafter, negative geotaxis performance was recorded, in seconds, daily.
• Eye opening: the first day of both eyes’ opening was recorded.
• Auditory startle reflex: the first day of the startle response to a clapping sound was recorded.
• Sensory eyelid reflex: the eyelid was gently touched with a cotton swab. The first day of eyelid contraction was recorded.

Rats are born altricial, so unable to perform complex behaviors. Reflex screening-level assessment appears the only testing available at very young ages (Moser, 2011; Nguyen et al., 2017).

### Immunohistochemistry

Three coronal hippocampal sections were cut −480 mm to −530 mm from Bregma along the rostrocaudal axis (Paxinos and Watson, 2007) of each of four rats per group (Saraceno et al., 2016; Herrera et al., 2018). On P21, rats were anesthetized (ketamine 40 mg/kg + xylazine 5 mg/kg, i.p.), and intracardially perfused with 4% paraformaldehyde in 0.1 M phosphate buffer, pH = 7.4. Brains were dissected out and immersed in the same fixative solution at room temperature for 2 h, and in 0.1 M phosphate buffer, pH = 7.4 at 4°C overnight. Coronal hippocampal sections (50 μm thick) were obtained (Vibratome VT 1000 S, Leica Microsystems, Wetzlar, Germany). Immunohistochemistry was performed on free-floating sections under moderate shaking. Endogenous peroxidase activity was quenched using a 0.3% hydrogen peroxide solution in methanol. Non-specific labeling was blocked with 0.3% normal goat serum diluted in phosphate-buffered saline (PBS) at room temperature (RT) for 1 h. Samples were PBS-washed 5 times for 10 min and incubated with anti-microtubule-associated protein 2 (MAP-2; 1:250, polyclonal rabbit-IgG; Abcam), anti-phosphorylated high and medium molecular weight neurofilaments (pNF H/M; 1:500, monoclonal mouse-IgG; Millipore), or anti-glial fibrillary acidic protein (GFAP; monoclonal rabbit IgG, 1:200, Cell Marque, a Sigma-Aldrich Company) diluted in 0.3% normal goat serum in PBS for 48 h at 4°C. The following day, samples were PBS-washed 5 times for 10 min and incubated with horseradish peroxidase (HRP) biotinylated secondary antibody (Biotinylated anti-mouse-IgG, 1:500, Vector; Biotinylated anti-rabbit-IgG, 1:500, Vector) diluted in PBS at room temperature for 2 h. Then, samples were PBS-washed 5 times for 10 min and incubated with an avidin-biotinylated HRP complex (1:500, Dako) in PBS in darkness at RT for 1 h, followed by 5 washes with PBS 10 min. Finally, the sections were incubated at RT for 5 min in 0.05% diaminobenzidine (DAB, Sigma) diluted in Tris–HCl 0.05 M pH = 7.4, containing 0.03% H_2_O_2_ for signal detection. After several running water-washes, the sections were transferred to a dish with 1× PBS for mounting. Glass slides were dipped into 1× PBS and a fine paintbrush was used to coax the sections gently towards the slide. After 1-h drying at RT, a drop of mounting medium (1:1 PBS: glycerol) was added barely to cover the tissue-section, and the coverslip was gradually placed starting with one edge against the slide and slowly releasing the coverslip nicely to avoid air bubbles. Finally, a thin nail polish layer was placed to seal the coverslip perimeter of and left to dry at RT (Bachman, 2013; Potts et al., 2020). Samples were observed using a digital camera-coupled Leica microscope, under constant light and brightness/contrast conditions. The images were processed and analyzed using ImageJ software (Image J 1.41o, NIH, United States). Antibody dilutions and DAB chromogen development time were unique for each protein staining. The intensity was determined in a blind fashion, using a semi-quantitative 0 to +++score.

### Morphometric analysis

The percentage of immunopositive area for pNF H/M and MAP-2 was estimated by sampling 150 μm^2^ per photomicrograph (ImageJ 1.41o, NIH, United States). The number of GFAP immunoreactive astrocytes was estimated in the CA1 hippocampal stratum radiatum area using the optical dissector method (Howard and Reed, 1998) with total section thickness for dissector height (Hatton and von Bartheld, 1999) and a 55 × 55 μm counting frame. A total of 78 counting frames per animal was assessed. Section thickness was measured using a microscope stage-attached digital length measuring device (Heidenhain-Metro MT 12/ND221; Traunreut, Germany). Every cell nucleus of GFAP-immunoreactive cells observed by focusing down through the height of the dissector was counted. Counts were performed on coded sections. Stratum radiatum volume in CA1 was estimated using the point count method (Weibel, 1979). Determinations were made by triplicate (Herrera et al., 2018).

### Western blot

The animals were euthanized by decapitation at 21 days of age and whole brains were extracted from the skull (Chiu et al., 2007). Hippocampi were macroscopically dissected out and stored frozen at −80°C. For protein extraction, specimens were thawed, homogenized in ice-cold lysis buffer (10 mM Tris/HCl, pH = 7.4, 10 mM NaCl, 3 mM MgCl_2_, 0.1% Triton X-100, protease inhibitors), and centrifuged at 14,000 rpm at 4°C for 15 min. Supernatants were sampled and protein content was quantified by Bradford dosage in a 96-well plate assay using bovine serum albumin (BSA) as standard. Each lane was loaded with samples containing 90 μg total protein diluted in buffer (0.3 M Tris/HCl, pH 7, 50% glycerol, 5% SDS, 1 mM EDTA, 0.1% bromophenol blue). Mini-cell protean II (Bio-Rad, Richmond, CA, United States) was used for sodium dodecyl-sulfate polyacrylamide gel electrophoresis (SDS-PAGE). Samples were resolved in 12.5% polyacrylamide discontinuous gels under denaturing conditions (SDS-PAGE) in Tris-Glycine buffer containing 25 mM Tris, 192 mM glycine (Bio-Rad), and 0.1% SDS at constant 120 V for 90 min. After rinsing in buffer baths at room temperature, proteins were electrophoretically transferred to PDVF membranes (MACHEREY-NAGEL, Germany) using the Hoefer TE 70 semi-dry transfer unit (Amersham Biosciences) in Towbin buffer (25 mM Tris, 192 mM glycine, 20% v/v methanol, 0.1% SDS, pH = 8.3) at constant 200 mA current intensity for 2 h. Membranes were blocked with 5% non-fat milk powder and 1% BSA in Tris-buffered saline containing 0.05% Tween 20 and incubated at 4°C overnight with anti-microtubule-associated protein 2 (MAP-2; 1:1,000, polyclonal rabbit-IgG; Abcam), anti-pNF H/M(1:500, monoclonal mouse-IgG; Millipore) or anti-GFAP (monoclonal mouse-IgG, 1:1,000; Santa Cruz Biotechnology). Anti-glyceraldehyde-3-phosphate dehydrogenase (GAPDH, 1:1,000, rabbit-IgG, Sigma-Aldrich) was used as the loading control. Blots were rinsed and incubated with HRP-conjugated secondary antibody (1:3,000, Bio-Rad, Richmond CA, United States) for 1 h at room temperature. Immunoreactive bands were detected using an ECL Western blotting analysis system (clarity western ECL substrate, Bio-Rad). After scanning films, the optical density of protein bands was quantified (Gel Pro Analyzer 3.1.00.00, Media Cybernetics). Four replicates were used, and experiments were run in triplicate (Herrera et al., 2018). Four brains per group were examined in triplicates (Herrera et al., 2018).

### Statistical analysis

Results were expressed as mean ± SEM. Shapiro–Wilk and Levene’s tests were used to check for normal distribution and equality of variances. Results underwent a two-way analysis of variance (ANOVAs) with birth condition (CTL or PA) and treatment (VHI or PEA) as main factors. For repeated measure variables like daily body weight and reflexes performance, a two-way ANOVA with group (CTL-VHI, PA-VHI, CTL-PEA, and PA-PEA) and day number as the main factors was used. Two-tailed Student’s *t*-test, adjusted by the Bonferroni correction, was used for *post hoc* comparisons. *p*-Value ≤ 0.05 was considered statistically significant, e.g., the probability that the null hypothesis was correct and results were random (type I error, or false positive) was ≤5% (Graphpad Prism version 7.04).

## Results

### Body weight gain in the first 3 weeks of life

**FIGURE 1:**
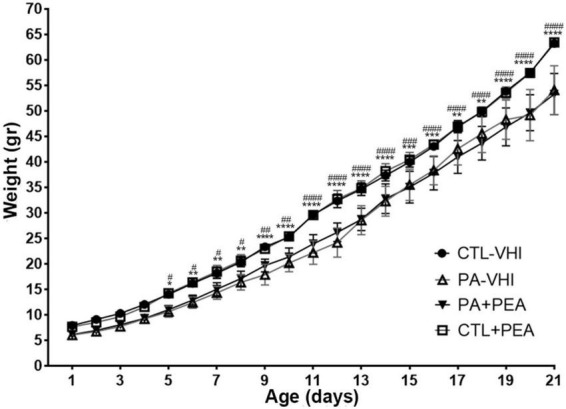
Body weight gain. Results are expressed as mean ± SEM. *p < 0.05, PA-VHI vs. CTL-VHI; **p < 0.01, PA-VHI vs. CTL-VHI; ***p < 0.001, PA-VHI vs. CTL-VHI; ****p < 0.0001, PA-VHI vs. CTL-VHI; ^#^*p* < 0.05, PA + PEA vs. CTL-VHI; ^##^*p* < 0.01, PA + PEA vs. CTL-VHI; ^###^*p* < 0.001, PA + PEA vs. CTL-VHI; ^####^*p* < 0.0001, PA + PEA vs. CTL-VHI. CTL-VHI, control rats treated with vehicle; PA-VHI, rats subjected to PA and treated with vehicle; PA + PEA, rats subjected to PA and treated with PEA; CTL + PEA, control rats treated with PEA. ***p < 0.001 PA-VHI vs. CTL-VHI.

Group (*F*_3,34_ = 18.51, *p* < 0.0001) and postnatal day (*F*_20,680_ = 3,938, *p* < 0.0001) were main sources of variation in daily body weight and showed interaction (*F*_60,680_ = 6.634, *p* < 0.0001). Starting on P5, PA-VHI rats’ daily body weight was lower than CTL-VHI rats’, but not different from PA + PEA rats at every time-point studied (Figure 1). All through the first weeks of life, CTL + PEA and CTL-VHI were indistinguishable based on body weight (Figure 1).

### Neurobehavioral development over the first 3 weeks of life

**FIGURE 2:**
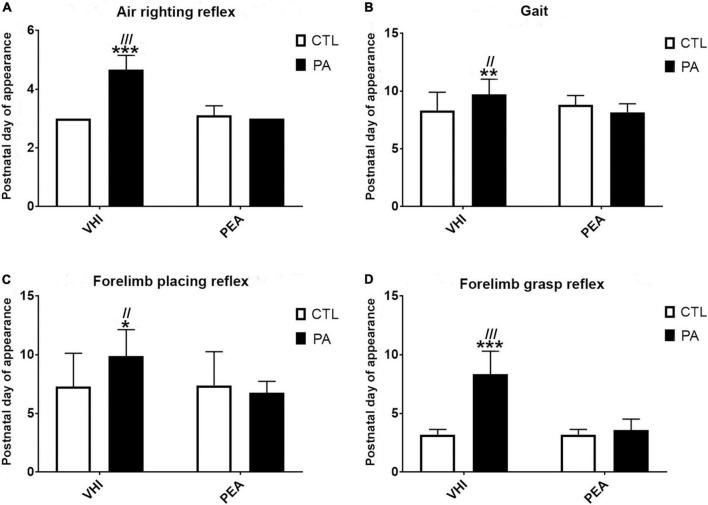
Neurobehavioral development. **(A)** Air-righting reflex. **(B)** Gait. **(C)** Forelimb placing reflex. **(D)** Forelimb grasp reflex. Results are expressed as mean ± SEM. *p < 0.05, PA-VHI vs. CTL-VHI; **p < 0.01, PA-VHI vs. CTL-VHI; ***p < 0.001, PA-VHI vs. CTL-VHI; ^//^*p* < 0.01, PA-VHI vs. PA + PEA; ^///^*p* < 0.001, PA-VHI vs. PA + PEA. CTL-VHI, control rats treated with vehicle; PA-VHI, rats subjected to PA and treated with vehicle; PA + PEA, rats subjected to PA and treated with PEA; CTL + PEA, control rats treated with PEA.

The appearance day of the air-righting reflex was affected by PA and PEA treatment (Figure 2A). Birth condition (*F*_1,66_ = 120.6, *p* < 0.0001) and treatment (*F*_1,66_ = 120.6, *p* < 0.0001) were main sources of variation, showing interaction (*F*_1,66_ = 157.5; *p* < 0.0001). The air-righting reflex appeared later in PA-VHI than in CTL-VHI rats (*p* < 0.0001), earlier in PA + PEA rats than in PA-VHI rats (*p* < 0.0001), while CTL + PEA and CTL-VHI rats showed no differences (*p* = 0.67) as *post hoc* analysis confirmed.

Perinatal asphyxia and PEA treatment affected gait appearance as well (Figure 2B). Two-way ANOVA reflected birth condition (*F*_1,71_ = 3.97, *p* = 0.04) and treatment (*F*_1,71_ = 14.56, *p* = 0.0003) were independent sources of variation (interaction: *F*_1,71_ = 1.97; *p* = 0.16). Gait appeared later in PA-VHI than in CTL-VHI rats (*p* = 0.003) and earlier in PA + PEA than in PA-VHI rats (*p* = 0.0009). No difference was found between CTL + PEA and CTL-VHI rats (*p* = 0.55).

Forelimb placing reflex appearance was affected by PA and PEA treatment (Figure 2C). Two-way ANOVA showed birth condition (*F*_1,67_ = 3.09, *p* = 0.008) and treatment (*F*_1,67_ = 7.12, *p* = 0.01) as main sources of variation, showing interaction (*F*_1,67_ = 8.21, *p* = 0.006). Forelimb placing appeared later in PA-VHI than in CTL-VHI rats (*p* = 0.008), and earlier in PA + PEA than in PA-VHI rats (*p* = 0.002), while CTL-VHI and CTL + PEA rats were indistinguishable (*p* = 0.99), as *post hoc* analysis confirmed. Birth condition (*F*_1,67_ = 103.2, *p* < 0.0001) and treatment (*F*_1,67_ = 77.49, *p* < 0.0001) also affected forelimb grasp reflex onset, showing interaction (*F*_1,67_ = 77.49, *p* < 0.0001) (Figure 2D). Forelimb grasp appeared later in PA-VHI than in CTL-VHI rats (*p* < 0.0001), earlier in PA + PEA than in PA-VHI rats (*p* < 0.0001), and concurrently in CTL + PEA and CTL-VHI rats (*p* > 0.9999).

**FIGURE 3:**
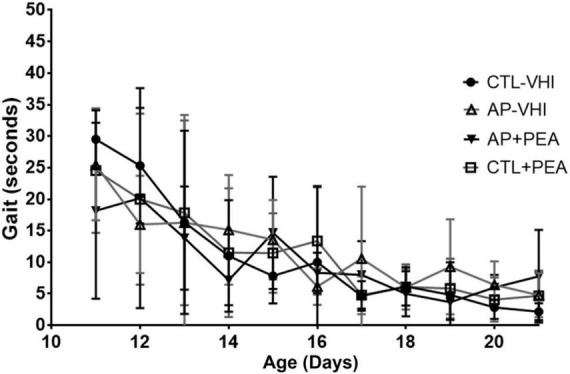
Gait performance. Results are expressed as mean ± SEM. CTL-VHI, control rats treated with vehicle; PA-VHI, rats subjected to PA and treated with vehicle; PA + PEA, rats subjected to PA and treated with PEA; CTL + PEA, control rats treated with PEA.

**FIGURE 4:**
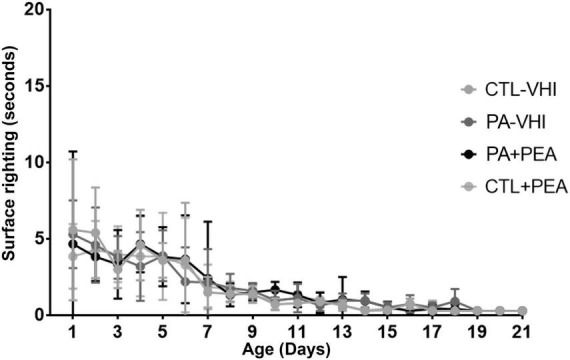
Surface righting performance. Results are expressed as mean ± SEM. Statistical analyses were conducted by two-way mixed ANOVA. CTL-VHI, control rats treated with vehicle; PA-VHI, rats subjected to PA and treated with vehicle; PA + PEA, rats subjected to PA and PEA treatment; CTL + PEA, control rats treated with PEA.

Regarding gait performance, two-way ANOVA revealed day number as the only source of variation (*F*_10,280_ = 21.38, *p* < 0.0001), while group had no effect (*F*_3,28_ = 0.1953, *p* = 0.8987) and interaction was null (*F*_30,280_ = 0.9828, *p* = 0.4961) (Figure 3). Likewise, two-way ANOVA showed day number as the only source of variation in surface righting performance (*F*_20,480_ = 28.16, *p* < 0.0001), which was unaffected by group (*F*_3,24_ = 0.1906, *p* = 0.9018) with null interaction (*F*_60,480_ = 0.4387, *p* > 0.9999) (Figure 4).

**FIGURE 5:**
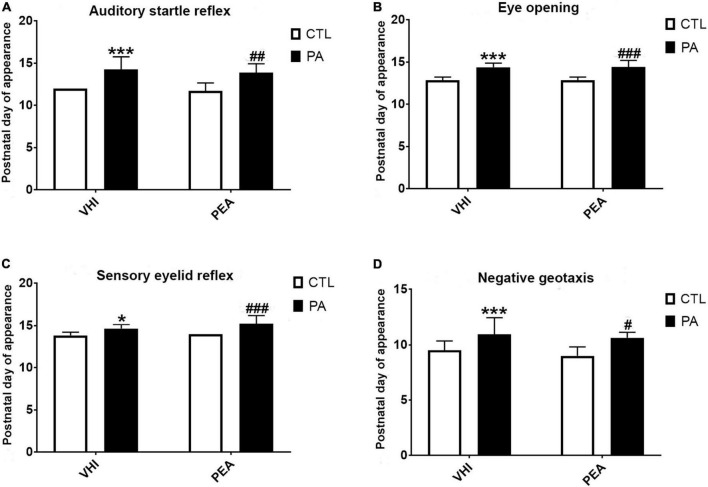
Postnatal day of reflexes appearance and neurological signs. **(A)** Auditory start reflex. **(B)** Eye opening. **(C)** Sensory eyelid reflex. **(D)** Negative Geotaxis. Bars and error bars represent mean ± SEM. *p < 0.05, PA-VHI vs. CTL-VHI; ***p < 0.001, PA-VHI vs. CTL-VHI; ^#^*p* < 0.05, PA + PEA vs. CTL-VHI; ^##^*p* < 0.01, PA + PEA vs. CTL-VHI; ^###^*p* < 0.001, PA + PEA vs. CTL-VHI. CTL-VHI, control rats treated with vehicle; PA-VHI, rats subjected to PA and treated with vehicle; PA + PEA, rats subjected to PA and treated with PEA; CTL + PEA, control rats treated with PEA.

The auditory startle reflex appearance was affected by PA but not by PEA treatment (Figure 5A). Two-factor ANOVA showed birth condition as a source of variation (*F*_1,45_ = 36.56, *p* < 0.0001) unlike treatment that was not (*F*_1,45_ = 0.86, *p* = 0.36) with no interaction (*F*_1,45_ = 0.02, *p* = 0.88). *Post hoc* analysis revealed a later onset in PA-VHI rats than in CTL-VHI rats (*p* = 0.0004), while PA-PEA and PA-VHI groups (*p* = 0.75) and CTL-PEA and CTL-VHI groups were indistinguishable (*p* = 0.97). Similarly, for eye opening onset (Figure 5B), two-way ANOVA showed birth condition as a source of variation (*F*_1,45_ = 66.51, *p* < 0.0001), unlike treatment (*F*_1,45_ = 0.004, *p* = 0.95), without interaction (*F*_1,45_ = 0.004, *p* = 0.95). *Post hoc* analysis confirmed a delay in PA-VHI compared with CTL-VHI rats (*p* < 0.0001) but neither between groups PA-PEA and PA-VHI (*p* = 0.99) nor CTL-PEA and CTL-VHI (*p* > 0.9999). The appearance of the sensory eyelid reflex (Figure 5C) depended on birth condition (*F*_1,45_ = 23.61, *p* < 0.0001), but not on treatment (*F*_1,45_ = 2.86, *p* = 0.09), devoid of interaction (*F*_1,45_ = 1.02, *p* = 0.32). *Post hoc* analysis revealed that this reflex appeared later in PA-VHI than in CTL-VHI rats (*p* = 0.04) while PA-PEA and PA-VHI (*p* = 0.07), and CTL-PEA with CTL-VHI groups were alike (*p* = 0.98).

**FIGURE 6:**
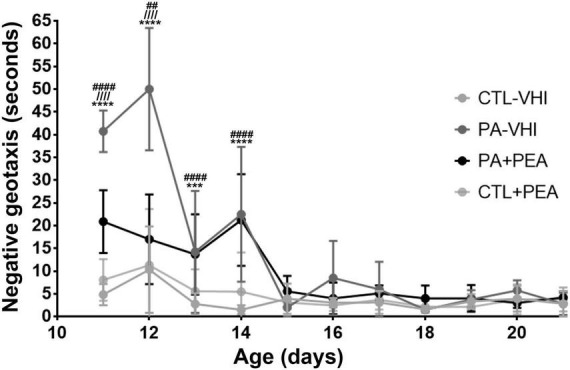
Negative geotaxis performance. Results are expressed as mean ± SEM. ***p < 0.001, PA-VHI vs. CTL-VHI; ****p < 0.0001, PA-VHI vs. CTL-VHI; ^////^*p* < 0.0001, PA-VHI vs. PA + PEA; ^##^*p* < 0.01, PA + PEA vs. CTL-VHI; ^####^*p* < 0.0001, PA + PEA vs. CTL-VHI. CTL-VHI, control rats treated with vehicle; PA-VHI, rats subjected to PA and treated with vehicle; PA + PEA, rats subjected to PA and treated with PEA; CTL + PEA, control rats treated with PEA.

Birth condition affected negative geotaxis onset (Figure 5D) (*F*_1,71_ = 45.44, *p* < 0.0001), while treatment did not (*F*_1,71_ = 3.02, *p* = 0.09), showing no interaction (*F*_1,71_ = 0.19, *p* = 0.66). Negative geotaxis appeared later in PA-VHI than in CTL-VHI rats, but concurrently in PA-PEA and PA-VHI (*p* = 0.81), and CTL-PEA and CTL-VHI rats (*p* = 0.39). Regarding negative geotaxis performance (Figure 6), two-way ANOVA showed group condition (*F*_3,44_ = 88.9, *p* < 0.0001) and postnatal day (*F*_10,440_ = 60.39, *p* < 0.0001) as sources of variation, showing interaction (*F*_30,440_ = 12.5, *p* < 0.0001). On P11, PA-VHI rats required more time than CTL-VHI rats did to complete the task (*p* < 0.0001), while PA-PEA rats were faster than PA-VHI rats (*p* < 0.0001). Groups CTL-PEA and CTL-VHI were indistinguishable (*p* = 0.23). Likewise, on P12, performance was slower in PA-VHI than in CTL-VHI rats (*p* < 0.0001). In addition, PA-PEA rats were faster than PA-VHI rats (*p* < 0.0001), and CTL-PEA and CTL-VHI rats were not different (*p* = 0.9437). On days P13 and P14, PA-induced negative geotaxis performance slowdown was still observed (*p* = 0.0003 and *p* < 0.0001, respectively). PA + PEA and PA-VHI (*p* = 0.9975 and *p* = 0.9751) and CTL-PEA and CTL-VHI groups were comparable (*p* = 0.3576 and *p* = 0.0958). Negative geotaxis performance was comparable in PA-VHI and CTL-VHI groups on P15 (*p* = 0.8994), P16 (*p* = 0.2116), P17 (*p* = 0.7314), P18 (*p* > 0.9999), P19 (*p* = 0.9988), P20 (*p* = 0.9175), and P21 (*p* > 0.9999).

### Cellular and biochemical modifications on postnatal day 21

**FIGURE 7:**
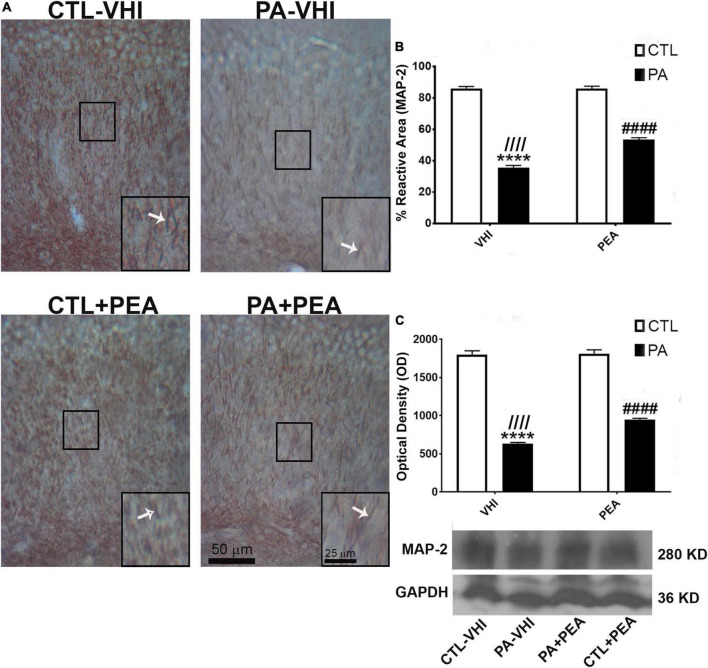
Microtubule-associated protein (MAP-2) immunostaining and protein expression in the rat hippocampus on P21. **(A)** Representative images of the stratum radiatum of CA1 hippocampal area immunostained for MAP-2. Scale bar: 50 μm. Marked rectangular areas in the main-image are shown magnified in the corresponding lower right margin. The white arrow indicates positive immunostaining for MAP-2. Scale bar: 25 μm. **(B)** Percentage of reactive area of MAP-2 positive dendrites. **(C)** Optical density of bands showing MAP-2 protein expression in Western blot. Results are shown as mean ± SEM. ****p < 0.0001, PA-VHI vs. CTL-VHI; ^////^*p* < 0.0001, PA-VHI vs. PA + PEA; ^####^*p* < 0.0001, PA + PEA vs. CTL-VHI. CTL-VHI, control rats treated with vehicle; PA-VHI, rats subjected to PA and treated with vehicle; PA + PEA, rats subjected to PA and treated with PEA; CTL + PEA, control rats treated with PEA.

Immunostaining for the specific dendrite marker MAP-2 allowed dendrite morphology examination (Figure 7A). Substantial fragmentation was observed in MAP-2 immunoreactive apical dendrites in the CA1 hippocampal area in the PA-VHI group compared with the CTL-VHI group that was partly attenuated in the PA-PEA group. Two-way ANOVA for MAP-2 reactive area results showed birth condition and treatment as main sources of data variation (*F*_1,56_ = 14,469, *p* < 0.0001; *F*_1,56_ = 658, *p* < 0.0001), having interaction (*F*_1,56_ = 677.5, *p* < 0.0001). *Post hoc* analysis confirmed a decrease in MAP-2 reactive area in PA-VHI compared with CTL-VHI rats (*p* = 0.0009) and, conversely, an increase in PA-PEA compared with PA-VHI rats (*p* = 0.0009) (Figure 7B). Western blot data analysis agreed with these results, confirming birth condition and treatment as major sources of MAP-2 protein expression variability (*F*_1,8_ = 2,235, *p* < 0.0001; *F*_1,8_ = 58, *p* < 0.0001), with interaction (*F*_1,8_ = 50, *p* < 0.0001). *Post hoc* analysis confirmed that MAP-2 protein expression was smaller in PA-VHI than in CTL-VHI rats (*p* = 0.0009), larger in PA-PEA than in PA-VHI rats (*p* = 0.0009), and not different in CTL-PEA and CTL-VHI rats (*p* = 0.99) (Figures 7B,C). Full scans of uncropped blots are presented in Supplementary Figure 1.

**FIGURE 8:**
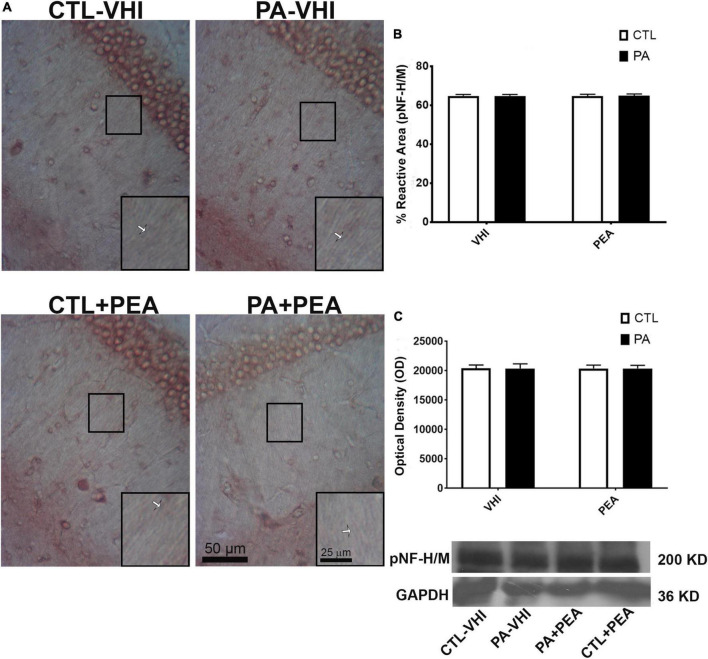
Phosphorylated high and medium molecular weight neurofilaments (pNF H/M) immunostaining and protein expression in the rat hippocampus on P21. **(A)** Representative images of the stratum radiatum of CA1 hippocampal area immunostained for pNF H/M. Scale bar: 50 μm. Marked rectangular areas in the main-image are shown magnified in the corresponding lower right margin. The white arrow indicates positive immunostaining for pNF H/M. Scale bar: 25 μm. **(B)** Percentage of reactive area of pNF H/M positive axons. **(C)** Optical density of bands showing pNF H/M protein expression in Western blot. Results are shown as mean ± SEM. PA-VHI, rats subjected to PA and treated with vehicle; PA + PEA, rats subjected to PA and treated with PEA; CTL + PEA, control rats treated with PEA.

Immunohistochemistry and Western blot analysis of pNF H/M reactive area and expression levels allowed axonal function evaluation. Neurofilaments’ aberrant phosphorylation is a hallmark of axonal degeneration (Grant and Pant, 2000; Sihag et al., 2007; Dale and Garcia, 2012; Chen et al., 2017) and is found in several human neurological diseases (Hirano, 1994; Mori et al., 1996; Bomont et al., 2000; Shepherd et al., 2002; Douglas-Escobar et al., 2010). Changes in immunoreactivity and phosphorylation status measured by Western blotting for pNF H/M give the pattern of PA-induced alterations in axonal functionality and are in agreement with our previous findings (Saraceno et al., 2010; Herrera et al., 2018). Figure 8A is a representative CA1 hippocampal *stratum radiatum* section immunostained for pNF H/M. Neither birth condition nor treatment were sources of variation according to two-way ANOVA (*F*_1,56_ = 0.19, *p* = 0.66; *F*_1,56_ = 0.06, *p* = 0.8, respectively; Figure 8B). In agreement with these findings, analysis of pNF H/M protein expression confirmed that birth condition and treatment were not sources of data variation (*F*_1,8_ = 0.0006, *p* = 0.98; *F*_1,8_ = 0.001, *p* = 0.97, respectively; Figure 8C). Full scans of uncropped blots are presented in Supplementary Figure 2.

**FIGURE 9:**
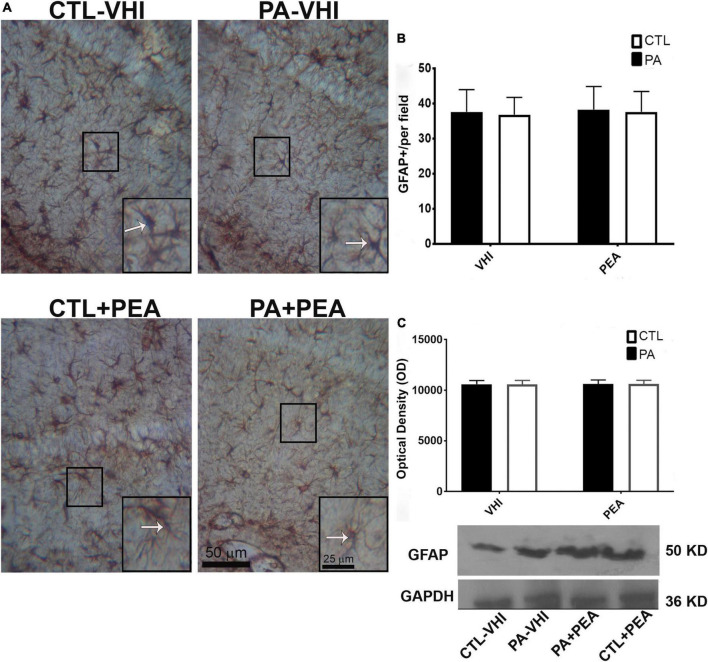
Glial fibrillary acidic protein (GFAP) immunostaining and protein expression in the rat hippocampus on P21. **(A)** Representative images of the stratum radiatum of CA1 hippocampal area immunostained for GFAP. Scale bar: 50 μm. Marked rectangular areas in the main-image are shown magnified in the corresponding lower right margin. The white arrow indicates positive immunostaining area for GFAP. Scale bar: 25 μm. **(B)** Percentage of reactive area of GFAP positive astrocytes. **(C)** Optical density of bands showing GFAP protein expression in Western blot. Results are shown as mean ± SEM. CTL-VHI, control rats treated with vehicle; PA-VHI, rats subjected to PA and treated with vehicle; PA + PEA, rats subjected to PA and treated with PEA; CTL + PEA, control rats treated with PEA.

Similar results were observed for glial response according to GFAP immunostaining data analysis (Figure 9A). The hippocampus and dentate gyrus, phylogenetically, of the oldest cortical areas, keep much of the radial orientation of their immature astroglial system (Eckenhoff and Rakic, 1984). In this region, a fusiform or rod-shaped and elongated morphology is observed (Zhou et al., 2019). All experimental groups showed a strikingly regular intense pattern of GFAP immunoreactivity in the hippocampus (Garcia-Segura et al., 1988; Hajós and Kálmán, 1989). Two-way ANOVA showed that the number of GFAP positive astrocytes was unrelated to either birth condition or treatment (*F*_1,56_ = 0.22, *p* = 0.64; *F*_1,56_ = 0.001, *p* = 0.97; Figure 9B). In agreement with these findings, GFAP protein expression was not affected by either birth condition or treatment (*F*_1,8_ = 0.007, *p* = 0.94; *F*_1,8_ = 0.01, *p* = 0.91; Figure 9C). Full scans of uncropped blots are presented in Supplementary Figure 3.

## Discussion

### Perinatal asphyxia-induced growth retardation and neurodevelopmental delay

In our study, PA for 19 min caused growth retardation, evidenced by low weight gain and severe neurodevelopmental delay by the first 3 weeks of life. The forelimb placing and grasp reflexes, resembling human palmar placing and grasp reflexes (Futagi et al., 2012; Nguyen et al., 2017), were delayed 3–5 days. Asphyctic animals had a 1.5-day delay in eye-opening, a 2-day delay in air-righting and auditory startle reflexes, and a 1-day delay in gait, negative geotaxis, and sensory eyelid reflex. Asphyctic rats showed slow negative geotaxis on P11 and P14 that normalized by the third week of life. These results extend earlier evidence after PA for 15 min, where forelimb placing and grasp reflexes were also affected the most. However, rats subjected to PA for 15 min (moderate PA) had body weight gain restored by the second week of life and normal eye opening (Kiss et al., 2009). Our findings show severe neurodevelopmental lag after 19 min of PA. Perinatal hypoxia-ischemia (HI) in Rice-Vanucci’s experimental model induced growth retardation (Fan et al., 2005, 2006; Lubics et al., 2005), delayed eye-opening (Fan et al., 2005, 2006; Romero et al., 2017) and grasping onset (Lubics et al., 2005), and slowed gait (Fan et al., 2005, 2006; Lubics et al., 2005), surface righting (Fan et al., 2005, 2006; Lubics et al., 2005), and negative geotaxis (Lubics et al., 2005), early signs of neurobehavioral dysfunction.

The neurodevelopmental delay observed over the first 3 weeks of life after PA precedes the alterations in exploratory activity, anxiety levels, and cognition on P30 (Barkhuizen et al., 2017). One month after PA, we found a decrease in rearing time (Herrera et al., 2018), i.e., vertical exploration in response to novelty, typically dependent on the integrity of the hippocampus (Lever et al., 2006). One-month-old rats subjected to an episode of 19–20 min of PA had reduced locomotion and rearing as well (Chen et al., 1995). Deficits in exploratory locomotion and increased anxiety in the open-field test were reported 1 month after severe PA (21 min). These 1-month-old asphyctic rats showed reduced exploratory locomotion on a squared area on P7 and slowed negative geotaxis on P14, and surface righting on P1 (Farfán et al., 2020).

### Perinatal asphyxia-induced hippocampal neuronal degeneration and dendritic alterations on postnatal day 21

Unlike studies on early growth and reflex development after PA (Kiss et al., 2009), we included morphological and biochemical analysis along with neurobehavioral testing. Instead of focusing on hippocampal oxidative stress or neuroinflammation (Farfán et al., 2020), we studied cytoskeletal modifications in CA1 neurons and the corresponding astrocytic response to extend our findings on P30 (Herrera et al., 2018). Neurobehavioral testing showed growth retardation and delayed reflexes over the first 3 weeks of life, and neuropathology examination on P21 confirmed CA1 hippocampal neurons’ vulnerability to severe PA (19 min). Besides signs of degeneration, these neurons showed decreased MAP-2 immunostaining and expression. MAP-2 seems an early biomarker of PA-induced neuronal injury, as observed in a birth-asphyxia piglet model (Lingwood et al., 2008), used to assess dendritic cytoskeletal dysfunction induced by HI (Malinak and Silverstein, 1996; Mink and Johnston, 2000; Sánchez et al., 2000; Zhu et al., 2003; Takita et al., 2004; Kühn et al., 2005; Graham et al., 2018). MAP-2 is phosphorylated by protein kinase A (PKA), an ATP-dependent enzyme. Then, PA-induced ATP reduction might explain MAP-2 decrease as phosphorylation might alter its susceptibility to proteolysis (Islam and Burns, 1981; Johnson et al., 1991; Grau et al., 1992; Sánchez et al., 2000; Ashworth et al., 2003).

In this work, the decreased hippocampal MAP-2 immunostaining and protein expression extend our findings on MAP-2 decreased level on P30 (Herrera et al., 2018), observed in the hippocampus as far as on P120 (Saraceno et al., 2010). In contrast, on P21 hippocampal pNF H/M level was stable and was not affected until P30 (Saraceno et al., 2010; Herrera et al., 2018). On P21, we have not found differences in either labeling intensity or the number of GFAP-positive astrocytes in the hippocampus. Once again, our results pose GFAP as a late biomarker of glial hippocampal damage following PA (Saraceno et al., 2016; Herrera et al., 2018), showing a significant increase 4 months following severe PA for 19 min (Saraceno et al., 2010). Likewise, clinical data suggests plain astrogliosis in a post-tertiary phase of damage (Douglas-Escobar and Weiss, 2015; Looney et al., 2015).

### Early neuroprotective effects of palmitoylethanolamide treatment

Palmitoylethanolamide (10 mg/kg) administered within the first hour of life, reversed the delay in the appearance of gait, air-righting, forelimb placing and grasp reflexes, and improved negative geotaxis performance on P11 and P12 in rats subjected to severe PA (19 min). PEA treatment reduced CA1 neuronal degeneration and cytoskeletal dendritic alterations on P21, as inferred from MAP-2 immunostaining and protein expression. Neuroprotection by PEA treatment against MAP-2 deficit and early neuromotor dysfunction has been observed in experimental neurodegeneration. PEA blunted Aβ42-induced reduction in MAP-2 labeling in degenerating neurons *in vitro* (Beggiato et al., 2018) and attenuated MAP-2 deficit in an *in vivo* Parkinson’s disease (PD) model (PEA 10 mg/kg) (Esposito et al., 2012). Therapeutic effects were reported for PEA (10 mg/kg) on limb locomotor rating scale over the first 8 days following experimental spinal cord injury (Genovese et al., 2008). Likewise, PEA prevented short-term limb weakness and altered gait in an experimental autoimmune encephalomyelitis model of multiple sclerosis (Rahimi et al., 2015).

Neuroprotective effects of PEA are mediated by peroxisome proliferator-activated receptor-alpha (PPAR-α) activation (Lo Verme et al., 2005) according to experimental evidence regarding PD, Alzheimer’s disease, traumatic brain injury and several neuropsychiatric disorders (Skaper et al., 1996; Genovese et al., 2008; Ahmad et al., 2012; D’Agostino et al., 2012; Esposito et al., 2012; Di Cesare Mannelli et al., 2013; Coppola and Mondola, 2013, 2014; Scuderi et al., 2014). The molecular mechanisms underlying PEA neuroprotective action *via* PPAR-α activation are still unknown. In PA, increased intracellular Ca^2+^ concentration and sustained endoplasmic reticulum (ER) stress may lead to calpain activation, with excess substrates’ degradation (Chohan et al., 2006; French et al., 2006). In this context, neuroprotection by PEA in experimental PA could result from decreased calpain activity *via* PPAR-α activation, reducing MAP-2 degradation, keeping cytoskeleton integrity. Calpain activity reduction by PPAR-α activation might be associated with decreased ER stress. However, understanding the molecular mechanism whereby PPAR-α activation reduces ER stress and stabilizes MAP-2 requires further research.

## Conclusion

Treatment with PEA (10 mg/kg) within the first hour of life attenuated neurodevelopmental delay in rats subjected to severe PA (19 min), reducing neurodegeneration and MAP-2 deficit in CA1 neurons on P21. Involved in the pathogenesis of several NDDs (Lasser et al., 2018), dendritic protein MAP-2 appears as an early marker of PA-induced hippocampal damage and a novel target for PEA-mediated neuroprotection. The therapeutic properties of this endogenous amide in NDDs have gathered evidence as case reports (Antonucci et al., 2015), experimental rodent models (Cristiano et al., 2018), randomized clinical trials (Khalaj et al., 2018), and comparative studies on animals and humans (Bertolino et al., 2017). Clinical research showed a high-safety profile for PEA (Steels et al., 2019). Therefore, PEA seems to be a promising neuroprotective agent against PA. Further studies should clarify the molecular mechanisms underlying PEA effects and help specify its precise indications.

## Data availability statement

The original contributions presented in this study are included in the article/Supplementary material, further inquiries can be directed to the corresponding author.

## Ethics statement

The animal study was reviewed and approved by the Institutional Animal Care and Use Committee of the University of Buenos Aires (CICUAL#4091/04).

## Author contributions

MH: conceptualization, experimental execution, data acquisition, and writing original draft. LU: experimental execution, data analysis and discussion, and writing. TK: bibliographic research and data acquisition. NT-U, CK, and RK-F: data acquisition, and reading and commenting. JL: data acquisition, administration, and supervision. MO-L: writing revision, conceptual, structural, language editing, correction, and proofreading. FC: conceptualization, supervision, and funding acquisition. All authors contributed to the article and approved the submitted version.

## Abbreviations

PA: perinatal asphyxia
PEA: palmitoylethanolamide
P: postnatal day
MAP-2: microtubule-associated protein 2
pNF H/M: phosphorylated high and medium molecular weight neurofilament
GFAP: glial fibrillary acidic protein
NDDs: neurodevelopmental disorders
TH: therapeutic hypothermia
NAEs: N-acylethanolamides
AEA: anandamide
OEA: oleoylethanolamide
HI: hypoxia-ischemia
ATP: adenosine triphosphate
cAMP: 3 ′ : 5 ′ -cyclic monophosphate
PKA: protein kinase A
PKC: protein kinase C
PD: Parkinson’s disease
PPAR-α: peroxisome proliferator-activated receptor-alpha
ER: endoplasmic reticulum.
