# Corrigendum: Palmitoylethanolamide attenuates neurodevelopmental delay and early hippocampal damage following perinatal asphyxia in rats

**DOI:** 10.3389/fnbeh.2022.1115398

**Published:** 2022-12-19

**Authors:** Maria I. Herrera, Lucas D. Udovin, Tamara Kobiec, Nicolas Toro-Urrego, Carlos F. Kusnier, Rodolfo A. Kölliker-Frers, Juan P. Luaces, Matilde Otero-Losada, Francisco Capani

**Affiliations:** ^1^Centro de Investigaciones en Psicología y Psicopedagogía, Facultad de Psicología, Pontificia Universidad Católica Argentina, Buenos Aires, Argentina; ^2^Centro de Altos Estudios en Ciencias Humanas y de la Salud, Universidad Abierta Interamericana, Consejo Nacional de Investigaciones Científicas y Técnicas, Buenos Aires, Argentina; ^3^Instituto de Ciencias Biomédicas, Facultad de Ciencias de la Salud, Universidad Autónoma de Chile, Santiago, Chile

**Keywords:** PEA, palmitoylethanolamide, perinatal asphyxia, neuroprotection, hippocampal CA1 area, reflexes, neurodevelopmental disorder (NDD)

In the original article, there was an error in affiliations #3 and #4. Instead of “1. Centro de Investigaciones en Psicología y Psicopedagogía, Facultad de Psicología, Pontificia Universidad Católica Argentina, Buenos Aires, Argentina; 2. Centro de Altos Estudios en Ciencias Humanas y de la Salud, Universidad Abierta Interamericana, Consejo Nacional de Investigaciones Científicas y Técnicas, Buenos Aires, Argentina; 3. Facultad de Psicología y Psicopedagogía, Pontificia Universidad Católica Argentina, Buenos Aires, Argentina; 4. Departamento de Biología, Universidad Argentina John F. Kennedy, Buenos Aires, Argentina; 5. Instituto de Ciencias Biomédicas, Facultad de Ciencias de la Salud, Universidad Autónoma de Chile, Santiago, Chile,” it should be “1. Centro de Investigaciones en Psicología y Psicopedagogía, Facultad de Psicología, Pontificia Universidad Católica Argentina, Buenos Aires, Argentina; 2. Centro de Altos Estudios en Ciencias Humanas y de la Salud, Universidad Abierta Interamericana, Consejo Nacional de Investigaciones Científicas y Técnicas, Buenos Aires, Argentina; 3. Instituto de Ciencias Biomédicas, Facultad de Ciencias de la Salud, Universidad Autónoma de Chile, Santiago, Chile.”

The authors apologize for this error and state that this does not change the scientific conclusions of the article in any way. The original article has been updated.

