# Supplementary figures and images for: Palmitoylethanolamide attenuates neurodevelopmental delay and early hippocampal damage following perinatal asphyxia in rats
Source: Front Behav Neurosci. 2022 Aug 25;16:953157. doi: 10.3389/fnbeh.2022.953157 (PMC9452789; doi:10.3389/fnbeh.2022.953157)

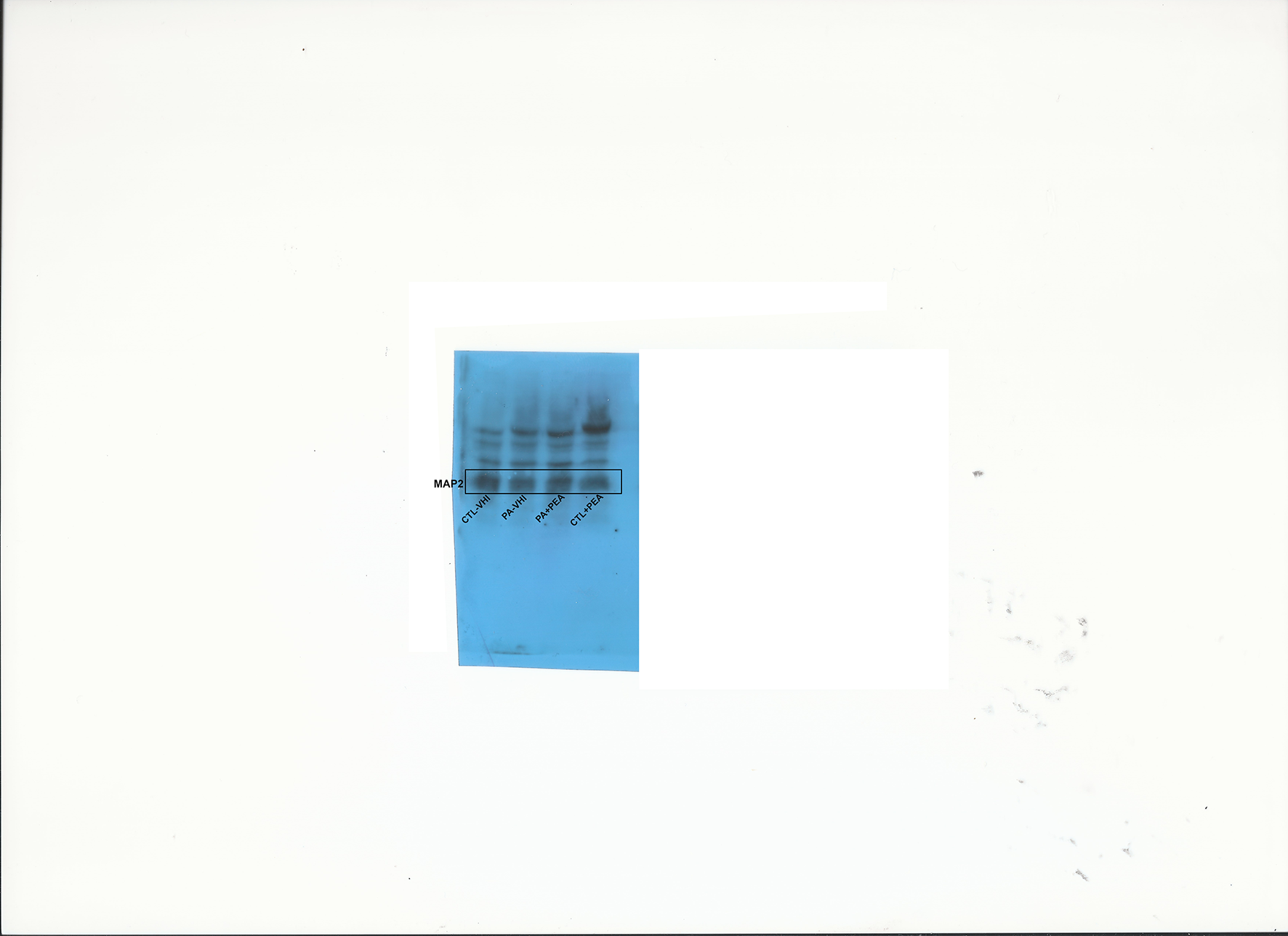

Supplement: Supplementary file 1 [file Image_1.TIF]

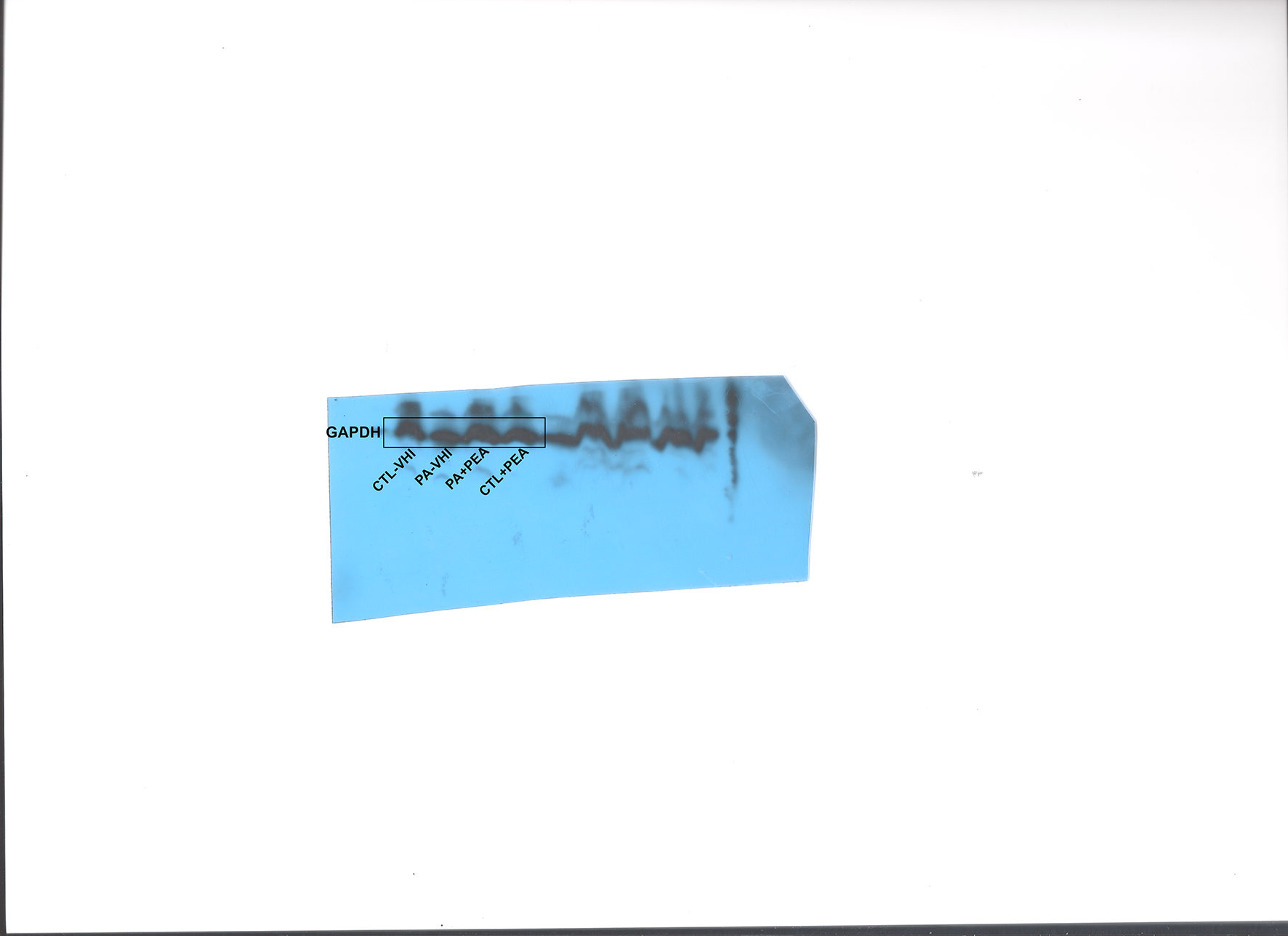

Supplement: Supplementary file 2 [file Image_2.TIF]

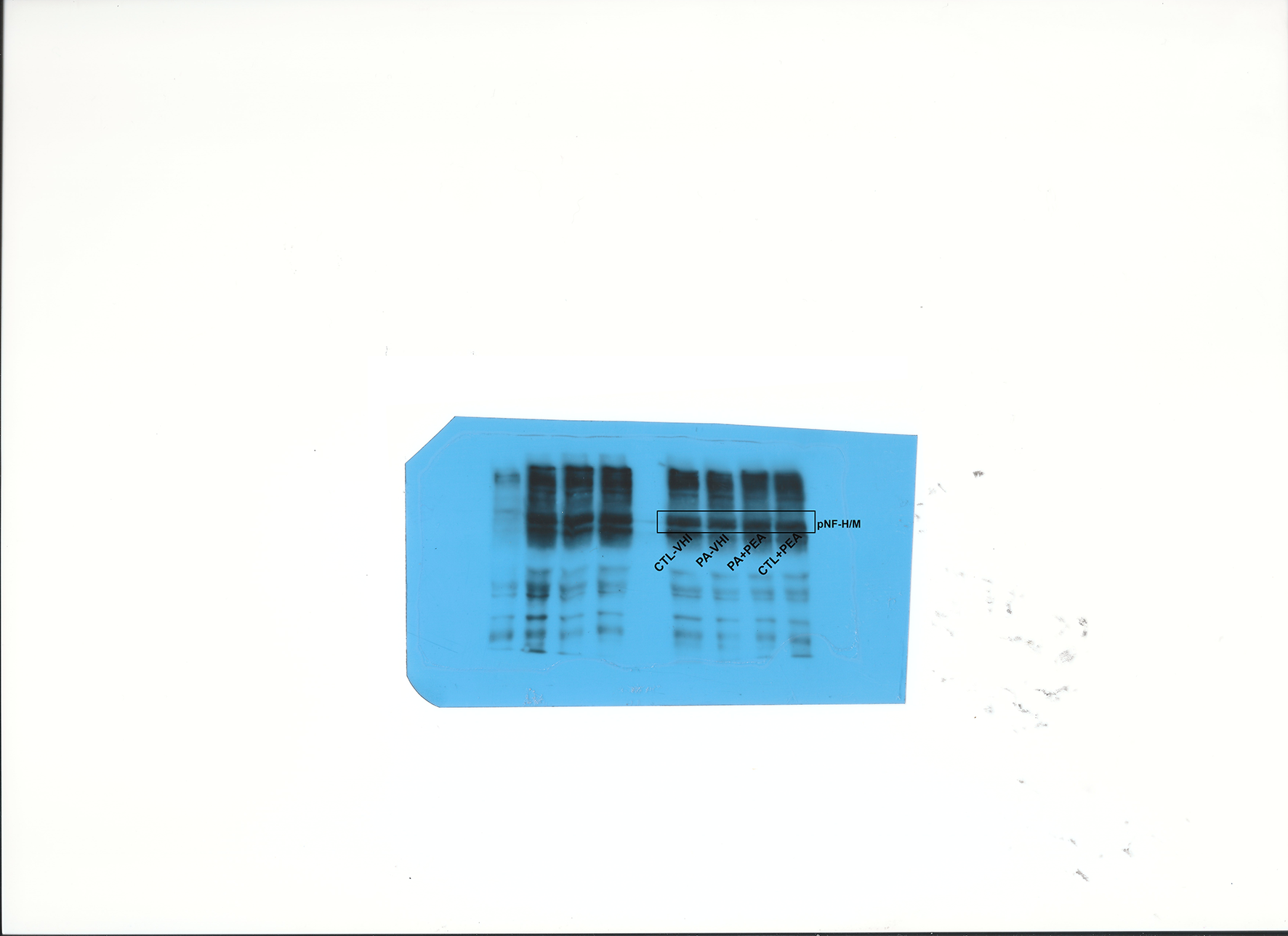

Supplement: Supplementary file 3 [file Image_3.TIF]

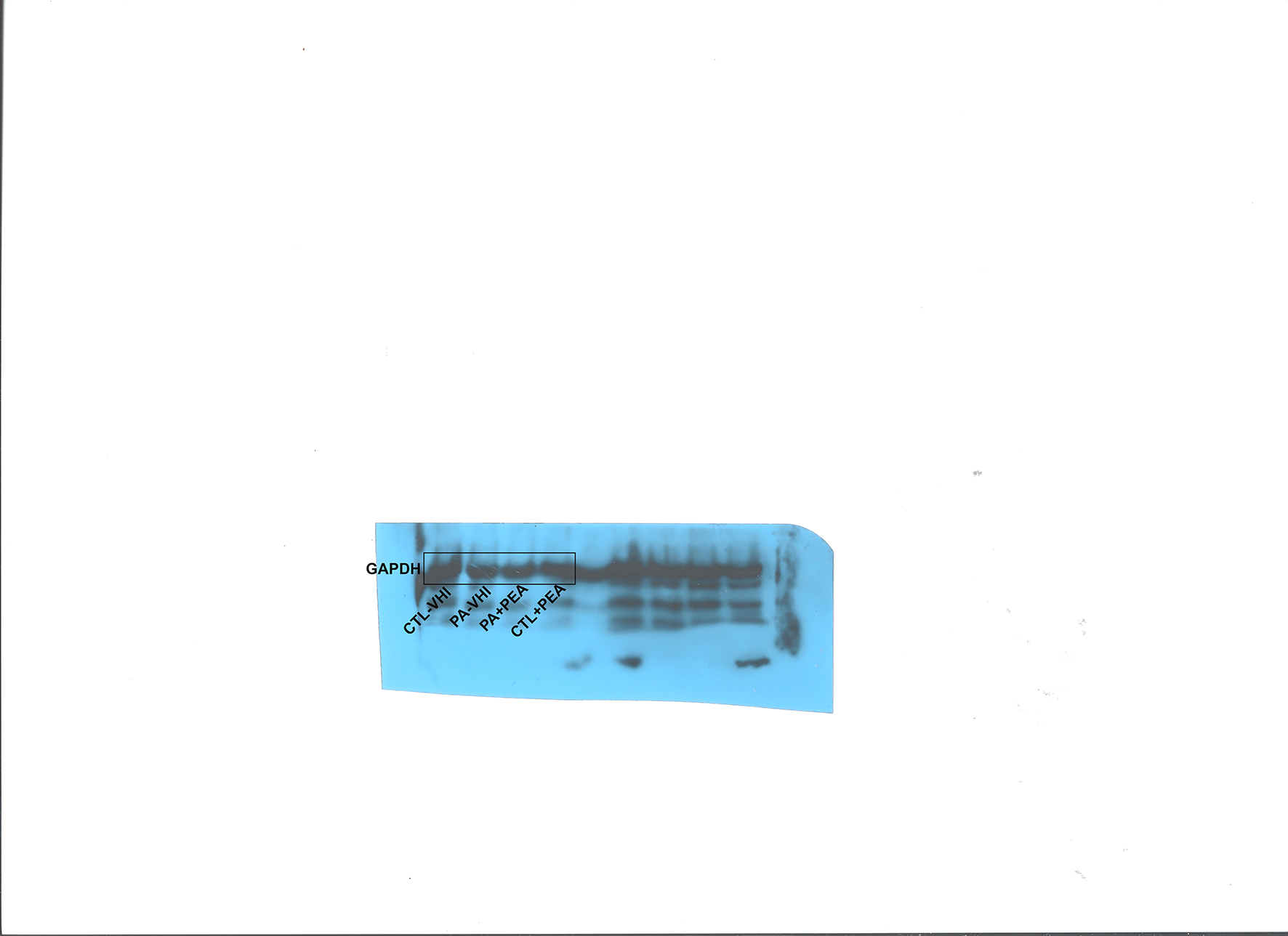

Supplement: Supplementary file 4 [file Image_4.TIF]

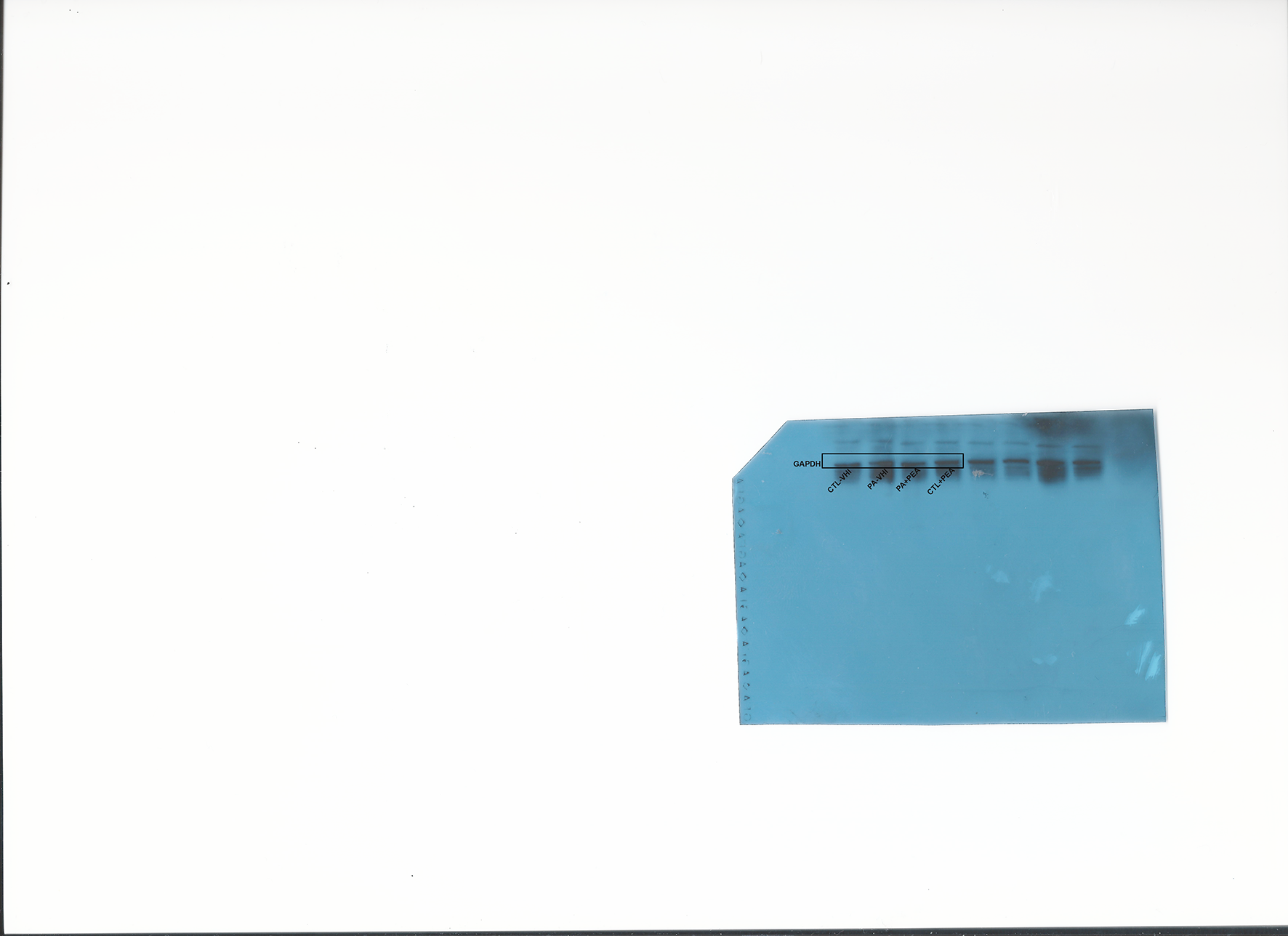

Supplement: Supplementary file 5 [file Image_5.TIF]

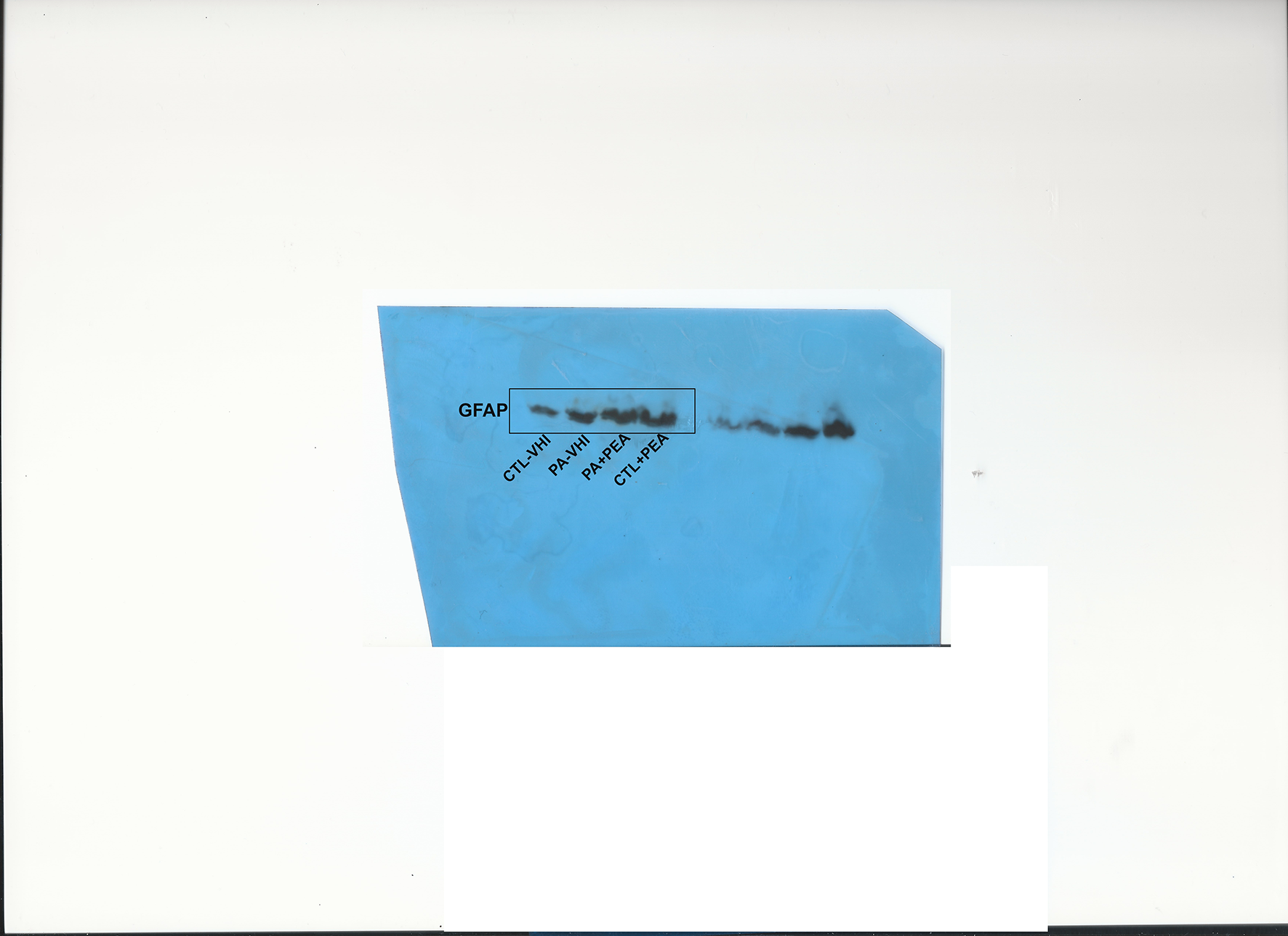

Supplement: Supplementary file 6 [file Image_6.TIF]
